# Paradoxical Effects of Rapamycin on Experimental House Dust Mite-Induced Asthma

**DOI:** 10.1371/journal.pone.0033984

**Published:** 2012-05-25

**Authors:** Karin Fredriksson, Jill A. Fielhaber, Jonathan K. Lam, Xianglan Yao, Katharine S. Meyer, Karen J. Keeran, Gayle J. Zywicke, Xuan Qu, Zu-Xi Yu, Joel Moss, Arnold S. Kristof, Stewart J. Levine

**Affiliations:** 1 Cardiovascular and Pulmonary Branch, National Heart, Lung, and Blood Institute, National Institutes of Health, Bethesda, Maryland, United States of America; 2 Laboratory of Animal Medicine and Surgery, National Heart, Lung, and Blood Institute, National Institutes of Health, Bethesda, Maryland, United States of America; 3 Pathology Core Facility, National Heart, Lung, and Blood Institute, National Institutes of Health, Bethesda, Maryland, United States of America; 4 Critical Care and Respiratory Divisions and Meakins-Christie Laboratories, Department of Medicine, McGill University Health Centre, Montreal, Quebec, Canada; University Hospital Freiburg, Germany

## Abstract

The mammalian target of rapamycin (mTOR) modulates immune responses and cellular proliferation. The objective of this study was to assess whether inhibition of mTOR with rapamycin modifies disease severity in two experimental murine models of house dust mite (HDM)-induced asthma. In an induction model, rapamycin was administered to BALB/c mice coincident with nasal HDM challenges for 3 weeks. In a treatment model, nasal HDM challenges were performed for 6 weeks and rapamycin treatment was administered during weeks 4 through 6. In the induction model, rapamycin significantly attenuated airway inflammation, airway hyperreactivity (AHR) and goblet cell hyperplasia. In contrast, treatment of established HDM-induced asthma with rapamycin exacerbated AHR and airway inflammation, whereas goblet cell hyperplasia was not modified. Phosphorylation of the S6 ribosomal protein, which is downstream of mTORC1, was increased after 3 weeks, but not 6 weeks of HDM-challenge. Rapamycin reduced S6 phosphorylation in HDM-challenged mice in both the induction and treatment models. Thus, the paradoxical effects of rapamycin on asthma severity paralleled the activation of mTOR signaling. Lastly, mediastinal lymph node re-stimulation experiments showed that treatment of rapamycin-naive T cells with *ex vivo* rapamycin decreased antigen-specific Th2 cytokine production, whereas prior exposure to *in vivo* rapamycin rendered T cells refractory to the suppressive effects of *ex vivo* rapamycin. We conclude that rapamycin had paradoxical effects on the pathogenesis of experimental HDM-induced asthma. Thus, consistent with the context-dependent effects of rapamycin on inflammation, the timing of mTOR inhibition may be an important determinant of efficacy and toxicity in HDM-induced asthma.

## Introduction

Rapamycin (Sirolimus, Rapamune®) is a macrolide product of Streptomyces hygroscopius that was initially discovered in a soil sample from Easter Island (Rapa Nui) in the early 1970s [1]. Rapamycin inhibits signaling by targeting the mammalian target of rapamycin (mTOR) with resultant immunosuppression and inhibition of cellular proliferation. Rapamycin is in clinical use for the prevention of kidney transplant rejection and has also been investigated as a treatment for tuberous sclerosis and lymphangioleiomyomatosis [2]. Similarly, rapamycin-eluting coronary artery stents have been developed to prevent re-stenosis [3], [4]. Rapamycin has also been proposed as a candidate treatment for asthma. Consistent with this, the ability of rapamycin-derivatives to inhibit asthma has been investigated in experimental animal models. The rapamycin derivative, 32-deoxorapamycin (SAR 943), was shown to be as effective as corticosteroids in inhibiting eosinophilic and lymphocytic airway inflammation, Th2 cytokine production, epithelial cell proliferation, goblet cell hyperplasia, and airway hyperreactivity in a murine model of ovalbumin (OVA)-induced asthma [5]. In contrast, intratracheal administration of 32-deoxorapamycin prior to a single OVA challenge in sensitized Brown-Norway rats, did not inhibit the number of bronchoalveolar lavage fluid eosinophils, lymphocytes or neutrophils, nor did it suppress airway hyperreactivity [6]. In another Brown-Norway rat model, repeated oral administration of 32-deoxyrapamycin to sensitized animals that had already begun to receive multiple OVA challenges, inhibited airway smooth muscle and epithelial cell proliferation and reduced the number of CD4+ T cells, but did not inhibit airway hyperreactivity or pulmonary eosinophilia [7]. Thus, previous studies report conflicting results regarding the utility of mTOR inhibitors for the treatment of asthma.

Here, we sought to define the role of mTOR signaling on the pathogenic manifestations of asthma using a clinically relevant house dust mite (HDM)-induced model of murine disease. We selected HDM to induce airway disease because it is an important environmental aeroallergen that has been identified as a risk factor for persistent asthma in human subjects [8], [9]. HDM has a heterogeneous composition that includes multiple proteins and lipopolysaccharide, which induces airway inflammation via both allergic and non-allergic pathways [10], [11], [12]. These pathways include toll-like receptor 4 signaling in airway epithelial cells, which activates both innate and adaptive immune responses [13]. We show that inhibition of mTOR signaling by rapamycin has paradoxical effects on the manifestations of HDM-induced asthma. Inhibition of mTOR signaling with rapamycin prior to the induction of asthma effectively suppressed airway inflammation, goblet cell hyperplasia and airway hyperreactivity, whereas inhibition of mTOR signaling in established asthma exacerbated airway inflammation and airway hyperreactivity, but did not modify HDM-induced increases in goblet cell hyperplasia.

## Methods

### Murine Models of Experimental House Dust Mite-induced Asthma

Female Balb/c mice were purchased from Jackson Laboratories (Bar Harbor, Maine). Asthma was induced by daily intranasal administration of HDM (*Dermatophagoides pteronyssinus*) (25 µg) or saline in a volume of 12 µl for 5 days each week [8]. In the induction model, mice received rapamycin (3 mg/kg) or vehicle (Phosal 50 PG®), by gavage daily, 5 days per week for 3 weeks concurrent with administration of nasal HDM. In the treatment model, mice received daily nasal HDM for 6 weeks and were treated with rapamycin (3 mg/kg) or vehicle (Phosal 50 PG®), by gavage daily, 5 days per week, during weeks 4 through 6. HDM was purchased from Greer Laboratories (Lenoir, NC), while the rapamycin oral solution was from Wyeth (Philadelphia, PA) and Phosal 50 PG® was from the American Lecithin Company (Oxford, CT). All experimental protocols (Protocols H-0210 and H-0244) were approved by the Animal Care and Use Committee of the National Heart, Lung, and Blood Institute. Results are representative of two independent experiments for each model.

### Bronchoalveolar Lavage and Lung Histopathologic Examination

Bronchoalveolar lavage (BAL) was performed utilizing three instillations of ice cold PBS (0.5 ml). Red blood cells present in BAL fluid (BALF) were lysed using ACK buffer (2 min at 4°C), followed by re-suspension of cells in 0.3 ml RPMI-1640 with 20% fetal bovine serum. The total number of BALF cells were counted using a hemocytometer. The differential cell counts were performed on Diff-Quick-stained cytospin slides (Siemens Healthcare Diagnostics, Deerfield, IL). For histopathological examination, lungs were inflated to a pressure of 25 cm of H_2_0 and fixed in 10% formalin for 24 h. Lungs were then dehydrated through gradient ethanol prior to embedding in paraffin. Sagittal sections were cut at a thickness of 5 mm and stained with hematoxylin and eosin or periodic acid Schiff (PAS). Representative histology images were selected by one of the authors who was blinded to the identity of the groups. Quantification of goblet cell hyperplasia was performed as previously described [14]. Briefly, all the airways present (large (conducting), medium (central), and small (distal)) within representative lung sections were analyzed and the number of airways containing PAS-positive cells were recorded. Goblet cell hyperplasia is presented as the percentage of airways containing PAS-positive cells. The number of airways inspected in each animal is also presented.

### Quantitative RT-PCR

Lungs were minced prior to storage in RNAlater® (Applied Biosystems Inc., Foster City, CA) at −70°C. Total RNA was subsequently isolated using the lipid tissue kit from Qiagen (Qiagen Inc, Valencia, CA) and on-column DNase treatment was performed using RNase-Free DNase from Qiagen. Reverse transcription was performed utilizing random hexamer primers and High Capacity cDNA Reverse Transcription Kit (Applied Biosystems, Foster City, CA). PCR was performed utilizing the TaqMan Universal PCR Master Mix and the following FAM dye-labeled Taqman® MGB probes; IL-4: Mm00445259_m1, IL-10: Mm00439614_m1, IL-13: Mm00434204_m1, IL-17a: Mm00439618_m1, Muc5Ac: Mm01276735_m1, Clca3: Mm00489959_m1, CCL7: Mm00443113_m1, CCL11: Mm00441238_m1, CCL17: Mm00516136_m1, CCL24: Mm00444701_m1 and 18S: Hs99999901_s1. One µg of cDNA was used as a template. and samples were amplified utilizing the 7500 Real Time PCR System running Sequence Detector version 2.1 software (ABI systems, Foster City, CA). Gene expression was quantified relative to the expression of 18S mRNA using the control sample as calibrator to calculate the difference in Ct values (ΔΔCt) and results are presented as relative mRNA expression.

### HDM-specific Re-stimulation of Mediastinal Lymph Node Cultures and Measurement of Th2 Cytokine Production

Mediastinal lymph nodes were removed, disrupted by gentle pressure with a syringe plunger and passed through a 100 µm strainer to yield single cell suspensions [15]. Following lysis of red blood cells with ACK buffer, mediastinal lymph node cells were suspended in RPMI 1640 medium containing 10% fetal calf serum, penicillin (50 units/ml), streptomycin (50 µg/ml), and L-gluamine (2 mM) and 4×10^6^ cells per well were cultured in 96-well plates with “U”-shaped bottoms. Cells were stimulated with or without HDM (100 µg/ml) and with or without rapamycin (10 nM) (Sigma-Aldrich, St. Louis, MO). After 96 hours, medium was collected and analyzed for cytokines by sandwich ELISAs with a limit of sensitivity of 15.6 pg/ml for IL-4 and IL-17A, 31.25 pg/ml for IL-5 and 62.5 pg/ml for IL-13 (R & D Systems, Minneapolis, MN).

### Measurement of Plasma IgE

Total plasma IgE was measured using an OptEIA™ kit (BD Biosciences Pharmingen, San Diego, CA). Total plasma IgG1 was measured using an ELISA Quantitation Set from Bethyl Laboratories, Inc. (Montgomery, TX), whereas total plasma IgG2a was measured using an ELISA Set from BD Biosciences Pharminogen (San Diego, CA).

### Detection and Quantification of Protein Levels

Whole lungs were removed en bloc, snap frozen in liquid nitrogen, and stored at −80°C prior to protein extraction. Lungs were mechanically disrupted using a Brickman mechanical homogenizer in homogenization buffer (20 mM Tris pH 8.0, 0.5% Nonidet P-40, 1 mM phenylmethanesulphonylfluoride, 50 mM NaF, 1 µg/ml aprotinin, 1 µg/ml leupeptin, 100 µM sodium orthovanadate). Homogenates were snap frozen on dry ice, thawed, and cleared by centrifugation at 16,000×g for 30 min at 4°C. Supernatants were assayed for protein content by Bradford assay. Lung proteins (80 µg) were separated by SDS-PAGE and transferred to nitrocellulose membrane before immunoblotting with primary antibodies as indicated. Membranes were incubated with anti-rabbit or anti-mouse IgG horseradish peroxidase conjugated antibodies and developed using Super-Signal West Pico chemiluminescence detection kit (Pierce). Antibodies that react with S6, phospho-S6, Akt, phospho-Akt, STAT6 and phospho-STAT6 were from Cell Signaling Technology, Inc. (Danvers, MA), whereas the antibody that reacted with ß-actin was from Sigma-Aldrich (St. Louis, MO).

### Measurement of Airway Hyperreactivity

Mice were anesthetized with ketamine and xylazine and a 19 gauge beveled metal catheter was inserted into the trachea. Mice were mechanically ventilated with a tidal volume of 0.2 ml at 2 Hz, while PBS or increasing doses of methacholine (0, 2.5, 5, 7.5 and 10 mg/ml) were administered by nebulization. Airway resistance was directly measured utilizing an Elan RC Fine Pointe system (Buxco Research Systems, Wilmington, N.C.). Airway resistance was recorded at 10 s intervals for 3 min and average values are presented as cm H_2_0/ml/s.

### Statistics

Results are presented as mean ± SEM. A one-way ANOVA with a Bonferroni's multiple comparison test was utilized for all analyses except for airway hyperreactivity experiments, which instead utilized a two-way ANOVA with a Bonferroni post-test test. A P value less than 0.05 was considered significant. Statistical analyses were performed with GraphPad Prism version 5.0a (Graphpad Software, Inc., La Jolla, CA).

## Results

### Paradoxical Effects of Rapamycin on Airway Inflammation in Murine Models of House Dust Mite-induced Asthma

**Figure 1:**
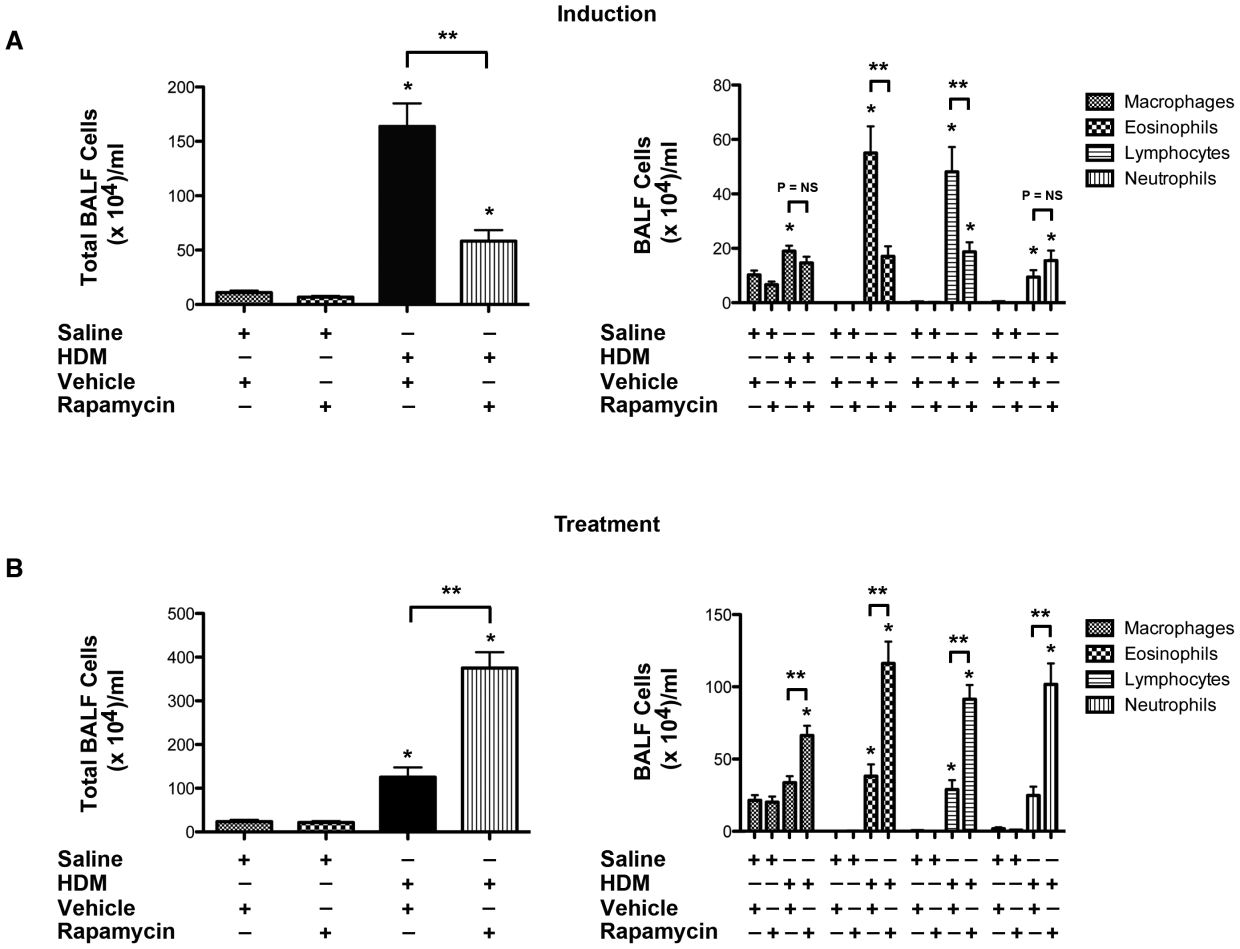
Paradoxical Effect of Rapamycin on Bronchoalveolar Lavage Fluid Inflammatory Cells in Induction and Treatment Models of House Dust Mite-induced Asthma. Balb/c mice received daily nasal challenges with HDM (25 µg) 5 days per week. In the induction model (Panel A), HDM challenges were initiated concurrent with rapamycin administration (3 mg/kg) by gavage 5 days per week for 3 weeks (n = 7–10 animals per group). In the treatment model (Panel B), HDM challenges were administered for 6 weeks and rapamycin administration was given during weeks 4 through 6 (n = 12–13 animals per group). * P<0.05 vs. Saline+Vehicle, ** P<0.001. Results are representative of two independent experiments.

**Figure 2:**
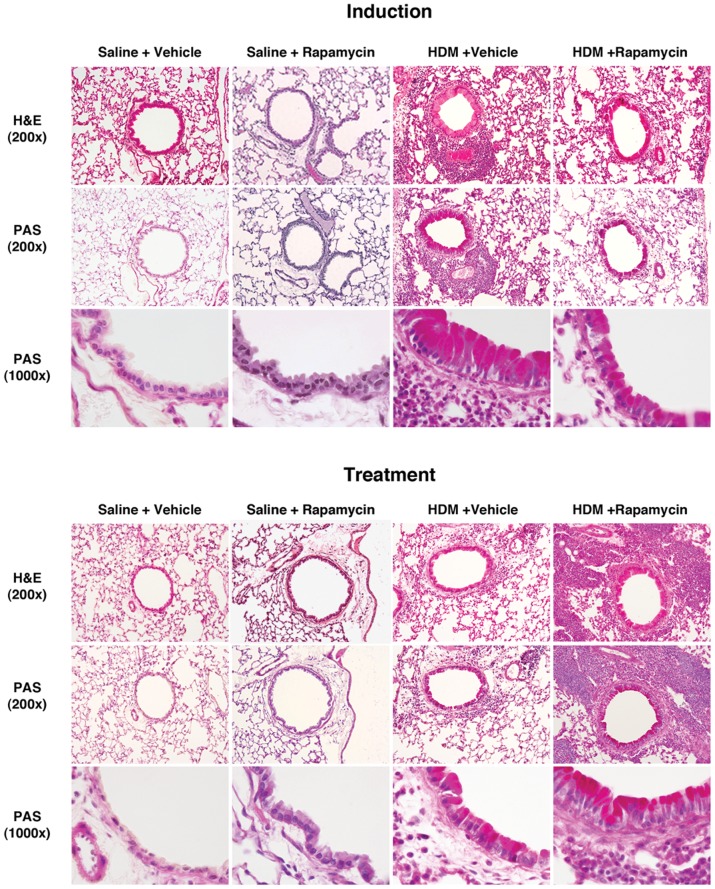
Paradoxical Effect of Rapamycin on Lung Histology in Induction and Treatment Models of House Dust Mite-induced Asthma. Histologic sections of lung were stained with hematoxylin and eosin (H & E) or periodic acid-Schiff (PAS) stains and images obtained at 200× or 1000×. Results are representative of 2 independent experiments.

To induce airway disease, Balb/c mice received daily nasal administration of HDM (25 µg) for 5 days per week. In the induction model, mice received rapamycin or vehicle coincident with the initiation of HDM administration for 3 weeks. In the treatment model, HDM challenges were performed for 6 weeks, whereas treatment with rapamycin or vehicle was administered during weeks 4 through 6 (Figure S1). In the induction model (Figure 1A), mice that had received rapamycin had a significant decrease in the total number of bronchoalveolar lavage fluid (BALF) inflammatory cells, specifically in the number of BALF eosinophils and lymphocytes. In contrast, in the treatment model (Figure 1B), rapamycin significantly increased the total number of BALF inflammatory cells, specifically in the number of eosinophils, lymphocytes, neutrophils and macrophages. As shown in Figure 2, the magnitude of peri-bronchial inflammatory cell infiltrates reflected the opposite effects of rapamycin on the number of BALF inflammatory cells in the induction and treatment models.

**Figure 3:**
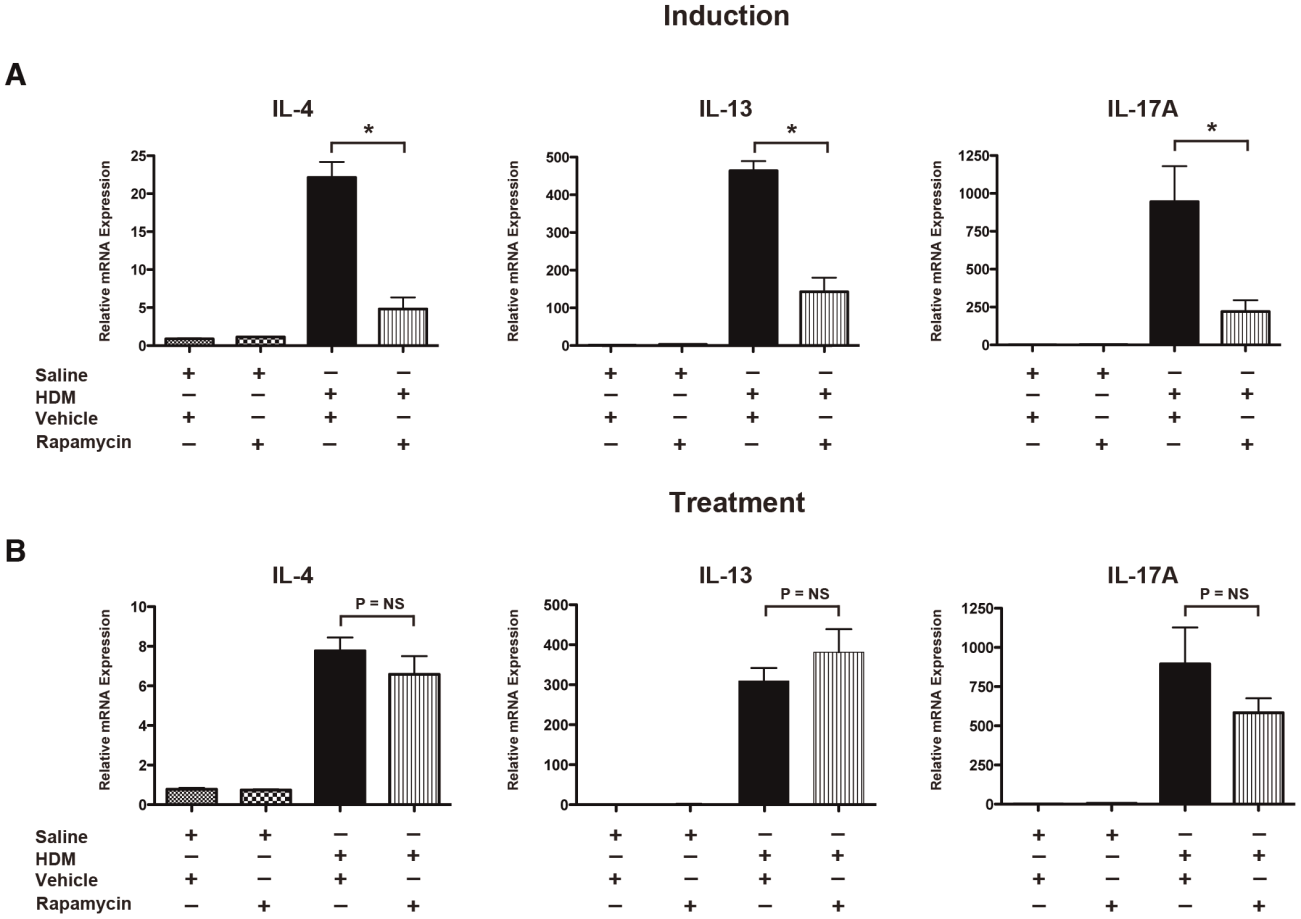
Paradoxical Effect of Rapamycin on Lung Th2 and Th17 Cytokine Expression in Induction and Treatment Models of House Dust Mite-induced Asthma. Quantification of lung mRNA levels for IL-4, IL-13, and IL-17A by qRT-PCR presented as relative mRNA expression. Results for the induction experiment are shown in Panel A (n = 6–8 animals per group, * P<0.05, HDM+Vehicle vs. HDM+Rapamycin), while results for the treatment experiment are shown in Panel B (n = 6–10 animals per group, * P<0.001). Results are representative of 2 independent experiments.

The pulmonary expression of Th2 and Th17 cytokines was assessed to investigate further the mechanisms mediating the differential effects of rapamycin in the induction and treatment models of HDM-induced asthma. As shown in Figure 3, administration of rapamycin prior to the induction of asthma significantly inhibited lung mRNA levels of canonical Th2 (IL-4 and IL-13) and Th17 (IL17A) cytokines. Treatment of established asthma with rapamycin, however, did not significantly alter the HDM-induced increases in lung mRNA levels of IL-4, IL-13 and IL-17A.

**Figure 4:**
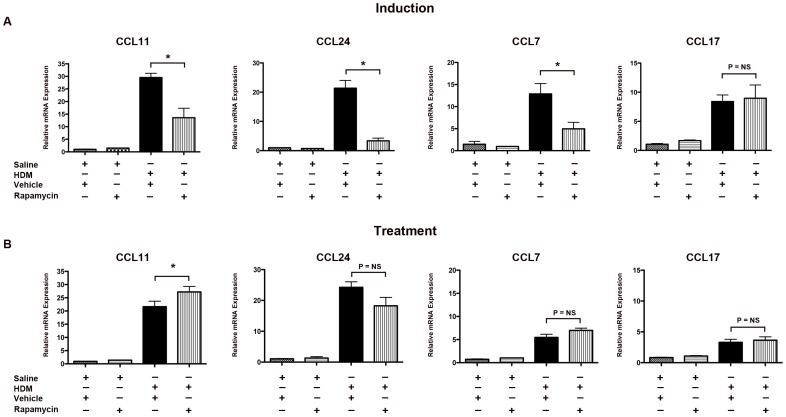
Paradoxical Effect of Rapamycin on Lung C-C Chemokine Expression in Induction and Treatment Models of House Dust Mite-induced Asthma. Quantification of lung mRNA levels for CCL11, CCL24, CCL7 and CCL17 by qRT-PCR presented as relative mRNA expression. Results for the induction experiment are shown in Panel A (n = 6 animals per group, * P<0.01), while results for the treatment experiment are shown in Panel B (n = 5–10 animals per group, * P<0.05). Results are representative of 2 independent experiments.

The effect of inhibition of mTOR on the expression of lung chemokines was also assessed. As shown in Figure 4, administration of rapamycin in the induction model significantly reduced the lung mRNA levels of key chemokines that mediate the chemotaxis of eosinophils and T cells to asthmatic lungs via binding to CCR3, including CCL11 (eotaxin-1), CCL24 (eotaxin-2) and CCL7 (MCP-3) [16], [17], [18]. Rapamycin treatment of established asthma, however, increased mRNA levels of CCL11 and did not affect the expression of CCL7 or CCL24. Rapamycin did not alter mRNA levels of CCL17 (TARC) in either the induction or treatment models of HDM-induced asthma.

These data demonstrate that administration of rapamycin prior to nasal HDM administration inhibits the induction of airway inflammation via a mechanism that involves the reduced expression of Th2- and Th17-type cytokines, as well as C-C chemokines. Conversely, treatment of established asthma with rapamycin increased both the number of BALF inflammatory cells, as well as lung mRNA levels of the C-C chemokine, CCL11.

### Paradoxical Effects of Rapamycin on Th2 T Cell Responses

**Figure 5:**
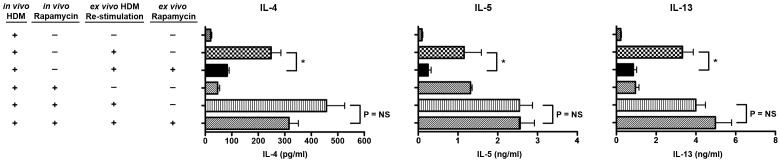
Effects of Rapamycin on Mediastinal Lymph Node Th2 Cytokine Production. Mediastinal lymph nodes from HDM-challenged mice that had or had not been treated with rapamycin concurrent with HDM stimulation for 3 weeks (induction model) were cultured *ex vivo* with or without HDM re-stimulation and/or rapamycin. The amount of IL-4, IL-5 and IL-13 released into the medium was quantified by ELISA (* P<0.05, n = 5–6). Results are representative of two independent experiments.

To investigate further the paradoxical effects of rapamycin on HDM-mediated airway inflammation, we used the induction model to assess the effects of *ex vivo* rapamycin on antigen-specific Th2 cytokine production by T cells isolated from the draining mediastinal lymph nodes of mice that had previously been sensitized to HDM *in vivo* with or without rapamycin administered by gavage. HDM re-stimulation of mediastinal lymph node cells recovered from mice that had been challenged with HDM for 3 weeks without *in vivo* rapamycin treatment showed a significant reduction in the production of the Th2 cytokines, IL-4, IL-5 and IL-13 (Figure 5). This result is consistent with a requirement for mTOR in Th2 cytokine production by HDM-sensitized T cells. Levels of the Th17 cytokine, IL-17A, were below the limit of detection of the assay. In contrast, HDM re-stimulation of mediastinal lymph node cells recovered from HDM-challenged mice that had been treated with rapamycin both *in vivo* and *ex vivo* showed no reduction in the production of IL-4, IL-5 or IL-13 as compared to cells that were only treated with rapamycin *in vivo*. Taken together, this shows that treatment of rapamycin-naive T cells with rapamycin decreases the antigen-specific production of Th2 cytokines, whereas prior exposure of T cells to HDM and rapamycin for 3 weeks *in vivo* makes them refractory to the suppressive effects of *ex vivo* rapamycin treatment on HDM-mediated Th2 cytokine production.

### Rapamycin has Divergent Effects on IgE Production in HDM-induced Asthma Disease

**Figure 6:**
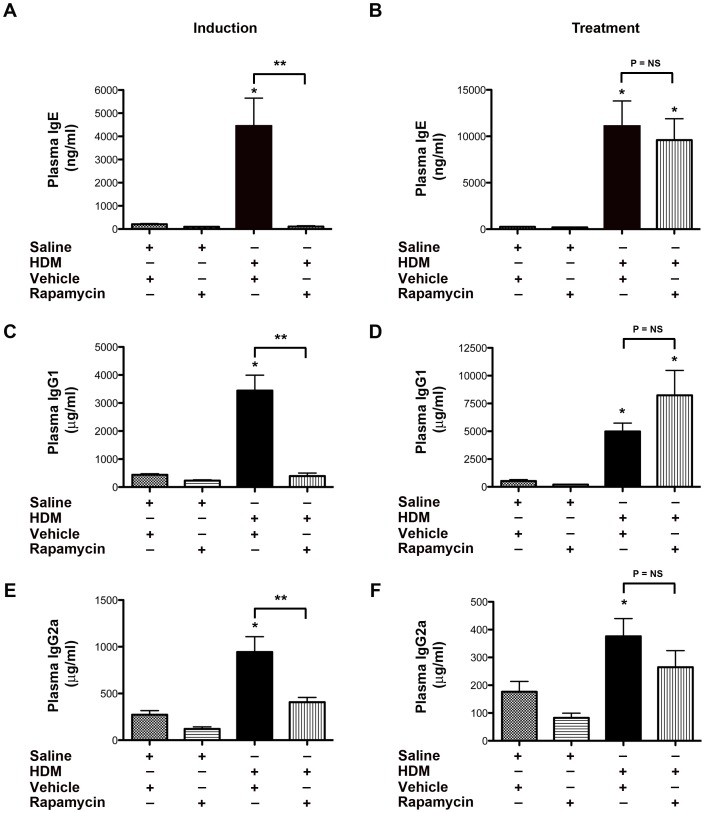
Paradoxical Effect of Rapamycin on Plasma Immunoglobulin Levels in House Dust Mite-induced Asthma. Plasma levels of IgE, IgG1 and IgG2a were quantified. Results for the induction experiment are shown in Panels A, C and E, while results for the treatment experiment are shown in Panels B, D and F (n = 8–20 animals per group, * P<0.05 vs. Saline+Vehicle, ** P<0.001).

Experiments were performed to assess whether rapamycin also has paradoxical effects on plasma IgE production. As shown in Figure 6, administration of rapamycin prior to the induction of HDM-induced asthma markedly attenuated plasma IgE, IgG1 and IgG2a levels, whereas treatment of established airway disease with rapamycin did not significantly modify plasma levels of IgE, IgG1 or IgG2a. These results suggest that inhibition of mTOR signaling with rapamycin inhibited sensitization to HDM allergens in the induction model, but did not reduce production of IgE, IgG1 or IgG2a once HDM sensitization had already occurred.

### Effect of Rapamycin on HDM-induced Goblet Cell Hyperplasia

**Figure 7:**
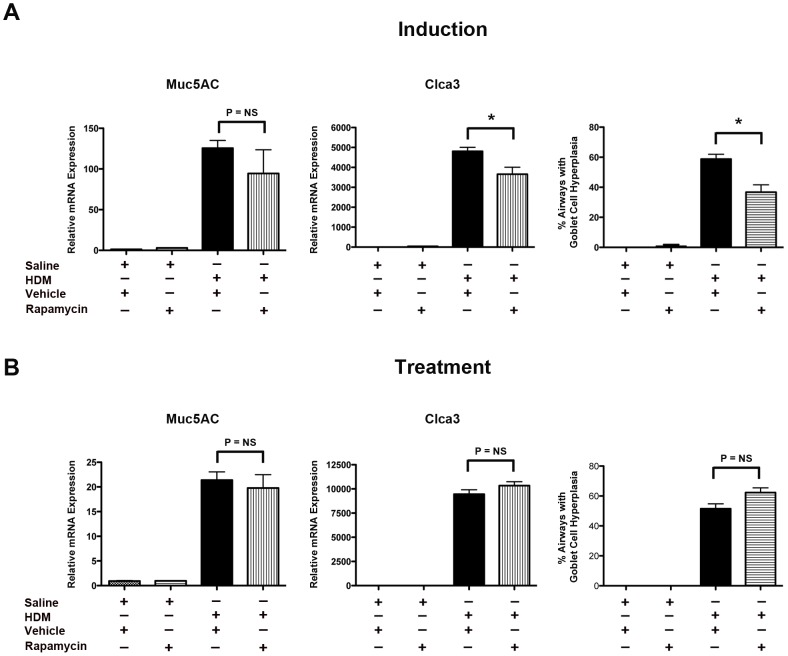
Paradoxical Effect of Rapamycin on Goblet Cell Hyperplasia in House Dust Mite-induced Asthma. Quantification of lung mRNA levels for Muc5AC and Clca3 by qRT-PCR are presented as relative mRNA expression. Results for the induction experiment are shown in Panel A (n = 6 animals per group, * P<0.01), while the results for the treatment experiment are shown in Panel B (n = 5–6 animals per group, P = NS). Results are representative of 2 independent experiments. Goblet cell hyperplasia is presented as the percentage of airways containing PAS-positive cells (n = 8–10 animals per group, * P<0.001). 35.3±0.6 airways were inspected in each mouse.

In the induction model, rapamycin administration was associated with small, but significant, decreases in the number of airways demonstrating goblet cell hyperplasia and Clca3 (chloride channel calcium activated 3) mRNA levels, whereas mRNA levels for Muc5AC (mucin 5, subtypes A and C) were not modified (Figure 7). In contrast, rapamycin did not modify goblet cell hyperplasia or mRNA levels of Muc5AC or Clca3 in the treatment model.

### Paradoxical Effect of Rapamycin on HDM-induced Airway Hyperreactivity

**Figure 8:**
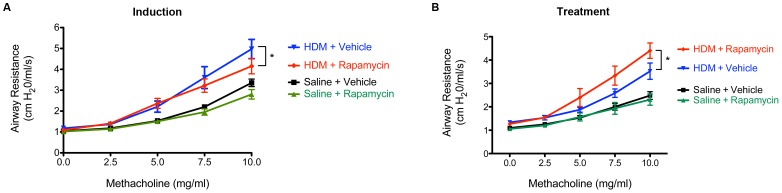
Paradoxical Effect of Rapamycin on Airway Hyperreactivity in House Dust Mite-induced Asthma. Airway resistance (cm H_2_0/ml/s) was directly measured following administration of increasing doses of nebulized methacholine. Results for the induction experiment are shown in Panel A (n = 10 animals per group, * P<0.05), while results form the treatment experiment are shown in Panel B (n = 9–10 animals per group, * P<0.05). Results are representative of 2 independent experiments.

The effect of rapamycin on HDM-induced airway hyperreactivity (AHR) was also assessed. As shown in Figure 8, administration of rapamycin prior to the induction of HDM-induced asthma resulted in a small, but significant decrease in AHR, whereas treatment of established HDM-induced airway disease with rapamycin was associated with a small, but significant increase in AHR.

### Temporal Association between mTOR Signaling and the Paradoxical Effects of Rapamycin in Induction and Treatment Models of HDM-induced Asthma

**Figure 9:**
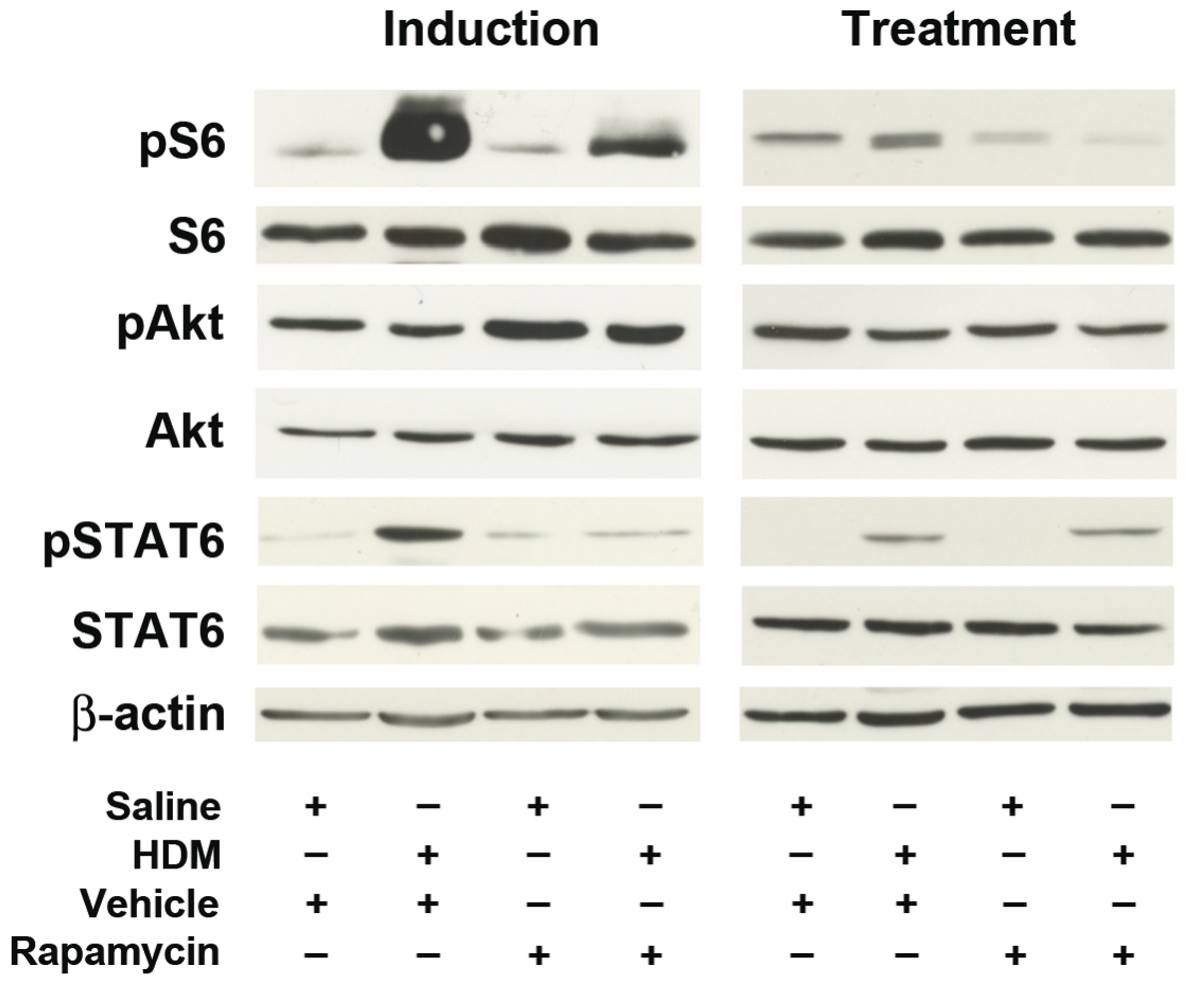
Rapamycin attenuates mTORC1 effectors. Phosphorylation of the mTORC1 effector, S6 (phospho-S6 Ser 235/236), or the mTORC2 effector, Akt (phospho-Akt S473), was assessed by Western blot analysis. The phosphorylation of STAT6 (phospho-STAT6 Y641) was determined as a control for activation of Th2 pathways. A representative blot from 5 experiments is shown.

Western blots of lung proteins were performed to identify the mechanism by which rapamycin mediates paradoxical effects on HDM-induced asthma. After 3 weeks in the induction model, phosphorylation of the S6 ribosomal protein was increased in the lungs of HDM-challenged mice as compared to saline-challenged mice (Figure 9). In contrast, after 6 weeks in the treatment model, S6 phosphorylation was no longer increased in HDM-challenged mice when compared to saline-challenged mice. In both the induction and treatment models, rapamycin attenuated S6 phosphorylation in HDM-challenged mice as compared to mice that received the vehicle control, which demonstrates inhibition of mTORC1 signaling. In contrast, Akt phosphorylation at serine 473 was neither increased by HDM-challenge nor inhibited by rapamycin, indicating that rapamycin did not inhibit TORC2 signaling. Lastly, although STAT6 phosphorylation was increased in HDM-challenged mice in both the induction and treatment models, it was only inhibited by rapamycin in the induction model.

## Discussion

Rapamycin is a specific and potent inhibitor of mTOR, a highly conserved and ubiquitous serine-threonine kinase that nucleates two distinct multi-protein mTOR complexes [19]. mTOR complex 1 (mTORC1) controls nutrient-sensitive protein synthesis and cell growth primarily via the initiation of translation and ribosomal biogenesis, whereas mTORC2 controls cytokinesis and cell survival via distinct effector kinases [20], [21]. Novel adaptor proteins define the functions of these mTOR complexes. mTORC1 contains the protein raptor and controls cell growth; mTORC2 contains the protein rictor and regulates cytoskeletal rearrangement. Rapamycin specifically blocks signaling via mTORC1 by binding to the immunophilin FK506-binding protein 1A (FKBP12), thereby disrupting the interaction between raptor and mTOR [1]. In contrast, mTORC2, which does not contain raptor, is thought to be resistant to direct inhibition by rapamycin. Multiple signaling pathways, such as antigen receptors, co-stimulatory molecules, growth factors, cytokines, hypoxia, cellular stress, low cellular energy levels and DNA damage regulate mTOR signaling. Activated mTORC1 phosphorylates at least two targets, S6 kinase 1 (S6K1) and the eukaryotic translation initiation factor-binding protein 1 (EIF4EBP1). Phosphorylation activates S6K1 and inhibits EIF4EBP1, with resultant enhancement of mRNA translation and cellular growth by augmentation of the cellular translational apparatus.

Inhibition of mTOR signaling by rapamycin has immunosuppressive effects on antigen-presenting cells and T cells, which thereby modulate adaptive immune responses. For example, rapamycin inhibits IL-4-dependent dendritic cell (DC) maturation, fms-like tyrosine 3 kinase ligand (Flt3L)-induced DC mobilization, as well as co-stimulatory molecule expression, pro-inflammatory cytokine production and T-cell allostimulation by DCs [1], [22]. Rapamycin attenuates DC-mediated antigen uptake and presentation via the inhibition of macropinocytosis and mannose receptor-mediated endocytosis [1], [23]. mTOR signaling also plays an important role in modulating the differentiation of effector T cells. mTOR activation is necessary for the differentiation and proliferation of Th1, Th2, and Th17 effector T cells, whereas T cells lacking mTOR differentiate into FOXP3^+^ regulatory T cells, which mediate immunological tolerance [24], [25]. Consistent with its therapeutic use as an immunosuppressant in organ transplant patients, rapamycin potently inhibits the expansion of T-cell populations [1]. Based upon its ability to inhibit the DC and effector T cell functions, rapamycin induces immune tolerance to solid organ transplants and is utilized clinically to prevent rejection in kidney transplantation [1]. mTOR inhibition also has effects on other immune cells that participate in the pathogenesis of asthma [1]. Rapamycin suppresses B cell responses and antibody production, inhibits neutrophil chemotaxis and prevents NKT cell proliferation.

mTOR signaling also plays an important role in airway smooth muscle and epithelial cell proliferation. mTORC1-related changes in the size, proliferation, and survival of smooth muscle or epithelial cells may contribute to hypertrophy and remodeling of the airway wall in asthmatics [26], [27]. Rapamycin prevents airway myocyte differentiation into a contractile phenotype via blockade of the mTORC1/p70 S6 kinase pathway, which may reduce the intrinsic contractile properties of airway smooth muscle [28]. Rapamycin also inhibits TGF-α-induced pulmonary fibrotic responses, which could contribute to sub-epithelial fibrosis and airway remodeling [29]. Similarly, mTOR signaling may modulate angiogenesis and lymphangiogenesis, both of which play important roles in asthma pathogenesis [30], [31]. Inhibition of mTOR blocks the synthesis of vascular endothelial growth factor (VEGF), an angiogenesis factor that induces an asthma phenotype in mice [32], [33]. mTOR physically interacts with the transcription factor HIF-1α, which regulates the expression of multiple angiogenesis genes [34]. Furthermore, mechanical strain of airway smooth muscle induces HIF-1α-dependent VEGF expression via mTOR and ERK pathways, which may thereby contribute to angiogenesis in asthma [32].

Based upon the important role of mTOR signaling in inflammatory and remodeling responses, we investigated whether rapamycin could be utilized to modify the pathogenic manifestations of asthma as well as provide new insights into disease pathogenesis. Here, we show that rapamycin has paradoxical effects depending on whether it is administered prior to the induction of HDM-induced asthma or as a treatment during the effector phase of HDM-induced asthma. Administration of rapamycin coincident with HDM exposure significantly attenuated eosinophilic and lymphocytic airway inflammation, which was mediated by the reduced expression of Th2- and Th17-type cytokines and C-C chemokines, as well as production of IgE, IgG1 and IgG2a. This is consistent with the role of mTOR in mediating the differentiation of CD4^+^ T cells into Th2 and Th17 subsets [24], [35]. Rapamycin also reduced the induction of AHR and goblet cell hyperplasia.

In contrast, treatment of established HDM-induced asthma with rapamycin augmented airway inflammatory responses, as indicated by significant increases in the number of BALF eosinophils, lymphocytes and neutrophils. Similarly, treatment of established HDM-induced asthma with rapamycin worsened AHR and did not reduce goblet cell hyperplasia or IgE production. Taken together, these results demonstrate that inhibition of mTOR signaling by rapamycin represents a “molecular switch” with divergent effects on asthma pathogenesis that are dependent upon the temporal relationship between rapamycin administration and allergen sensitization. When administered concurrent with HDM, rapamycin blocked allergic sensitization and the induction of the key manifestations of asthma, but when administered in the setting of established asthma, rapamycin exacerbated disease severity as evidenced by enhanced airway inflammation and AHR.

The mechanism underlying the paradoxical effects of rapamycin in the induction and treatment models of HDM-induced asthma may in part have reflected the temporal association between HDM challenge and activation of mTOR signaling pathways. Activation of mTORC1 signaling was assessed by phosphorylation of its downstream target, the S6 ribosomal protein. S6 phosphorylation was increased after 3 weeks of HDM challenge in the induction model, but returned to levels similar to those of saline-challenged mice at 6 weeks in the treatment model. Consistent with this finding, inhibition of mTOR signaling with rapamycin attenuated HDM-induced asthma only in the induction model when mTOR signaling was up-regulated, but not in the treatment model when mTOR signaling was no longer increased. Changes in STAT6 phosphorylation paralleled the effects of rapamycin on HDM-induced asthma. Furthermore, Akt phosphorylation was neither up-regulated by HDM challenge nor inhibited by rapamycin, which is consistent with the conclusion that mTORC2 signaling did not modulate HDM-induced asthma in this model.

We also found that rapamycin had paradoxical effects on Th2 cytokine production by T cells. The *ex vivo* treatment of mediastinal lymph node cells from HDM-challenged, rapamycin-naive mice with rapamycin attenuated the production of Th2 cytokines, which is consistent with the ability of rapamycin to inhibit the induction of allergen-mediated Th2-type inflammatory responses by HDM-sensitized T cells. In contrast, *ex vivo* rapamycin treatment of mediastinal lymph node cells from HDM-challenged mice that had already been exposed *in vivo* to rapamycin did not suppress Th2 cytokine production. This shows that prior rapamycin treatment renders mediastinal T cells refractory to the suppressive effects of rapamycin on Th2 cytokine production. Thus, these data are additional evidence supporting the context-dependent effects of mTOR inhibition on Th2 cytokine production.

Our findings are consistent with prior reports that showed paradoxical pro-inflammatory effects of rapamycin. For example, although rapamycin has immunosuppressive effects and is utilized clinically to prevent kidney transplant rejection, its use has been complicated by the development of a lymphocytic interstitial pneumonitis [36]. Glomerulonephritis and anemia secondary to a chronic inflammatory state have also been observed in patients treated with mTOR inhibitors, which is consistent with a pro-inflammatory role for rapamycin in some patients [37], [38], [39]. Similarly, in a murine model of cigarette smoke-induced lung inflammation, rapamycin decreased cigarette smoke-induced phosphorylation of NF-κappaB p65 [40]. In contrast, mice exposed to room air had an increase in BALF macrophages and neutrophils, NF-κappaB p65 phosphorylation and alveolar lung cell apoptosis. At the cellular level, mTOR can physically interact with NF-κappaB or STAT1, both of which are associated with airway inflammation in asthma. Furthermore, in dendritic cells and epithelial cells, reduced mTOR activity has been associated with enhanced NF-κappaB and STAT1 activity [41], [42], [43].

In conclusion, our results demonstrate that inhibition of mTOR signaling with rapamycin has paradoxical effects on the pathogenesis of HDM-induced asthma that are dependent upon the temporal relationship between rapamycin administration and the activation of mTOR signaling pathways. Rapamycin attenuated the manifestations of HDM-induced asthma after 3 weeks in the induction model when mTOR signaling was increased, but exacerbated HDM-induced asthma after 6 weeks in the treatment model, when mTOR signaling had returned to basal levels. Furthermore, our findings suggest that rapamycin might not represent an effective treatment approach for HDM-induced asthma.

## Supporting Information

Figure S1 **House dust mite (HDM) or saline was administered daily 5 days per week via an intranasal route for 3 weeks in the induction model (A) or 6 weeks in the treatment model (B).** Similarly, mice received rapamycin or vehicle by oral gavage 5 days per week for 3 weeks in the induction model (A) or during weeks 4 through 6 in the treatment model (B).
(TIF)
