# Supplementary figures and images for: Paradoxical Effects of Rapamycin on Experimental House Dust Mite-Induced Asthma
Source: PLoS One. 2012 May 25;7(5):e33984. doi: 10.1371/journal.pone.0033984 (PMC3368343; doi:10.1371/journal.pone.0033984)

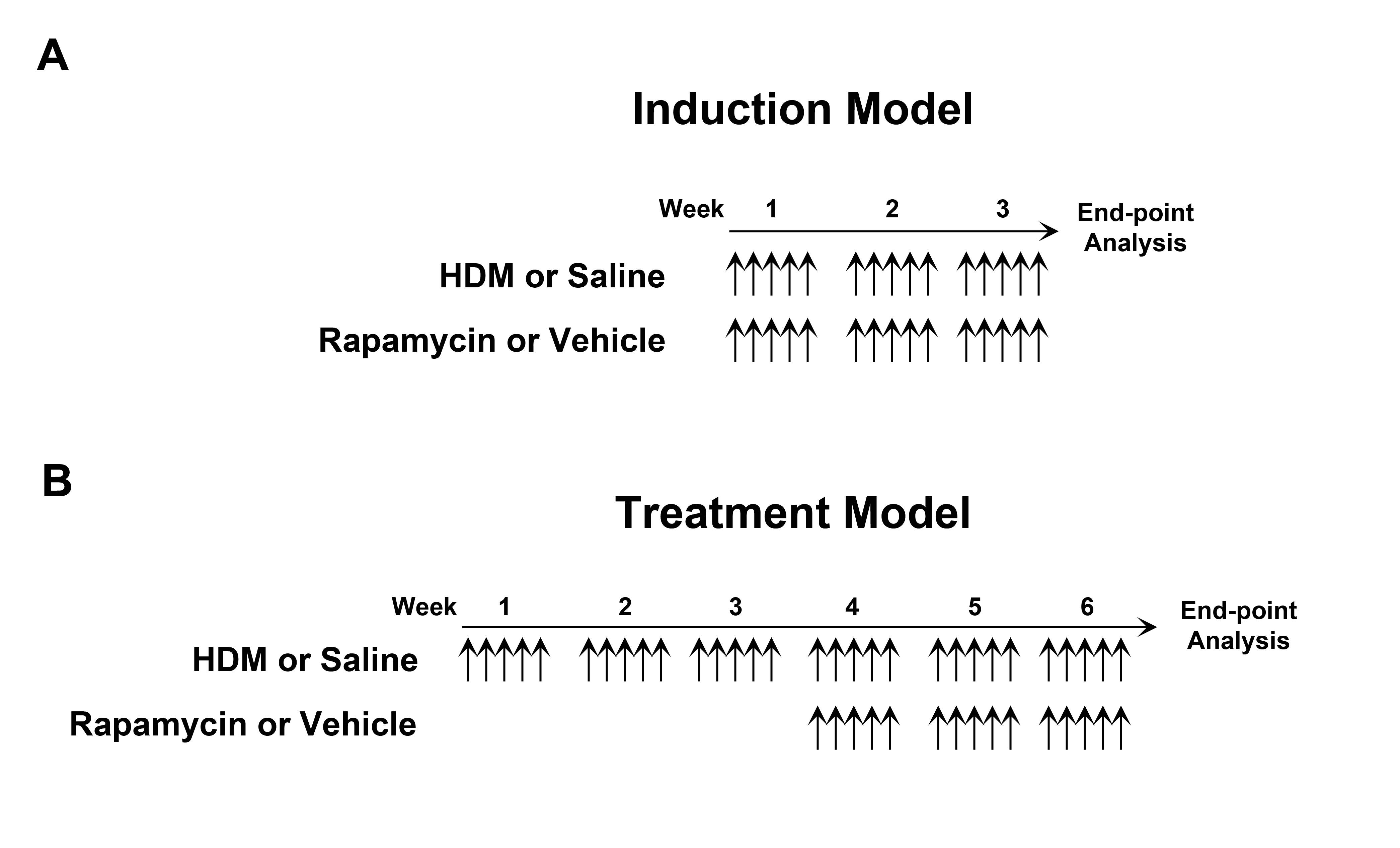

Supplement: Figure S1 — House dust mite (HDM) or saline was administered daily 5 days per week via an intranasal route for 3 weeks in the induction model (A) or 6 weeks in the treatment model (B). Similarly, mice received rapamycin or vehicle by oral gavage 5 days per week for 3 weeks in the induction model (A) or during weeks 4 through 6 in the treatment model (B). (TIF) [file pone.0033984.s001.tif]
